# The genome sequence of the Phoenix, *Eulithis prunata* (Linnaeus, 1758)

**DOI:** 10.12688/wellcomeopenres.19371.1

**Published:** 2023-04-25

**Authors:** Douglas Boyes, Peter W.H. Holland, University of Oxford and Wytham Woods Genome Acquisition Lab, Darwin Tree of Life Barcoding collective, Wellcome Sanger Institute Tree of Life programme, Wellcome Sanger Institute Scientific Operations: DNA Pipelines collective, Tree of Life Core Informatics collective, Darwin Tree of Life Consortium

**Affiliations:** 1 UK Centre for Ecology & Hydrology, Wallingford, England, UK; 2 University of Oxford, Oxford, England, UK

**Keywords:** Eulithis prunata, the Phoenix, genome sequence, chromosomal, Lepidoptera

## Abstract

We present a genome assembly from an individual male *Eulithis prunata* (the Phoenix; Arthropoda; Insecta; Lepidoptera; Geometridae). The genome sequence is 263.1 megabases in span. Most of the assembly is scaffolded into 30 chromosomal pseudomolecules, including the Z sex chromosome. The mitochondrial genome has also been assembled and is 15.9 kilobases in length. Gene annotation of this assembly on Ensembl identified 16,023 protein coding genes.

## Species taxonomy

Eukaryota; Metazoa; Ecdysozoa; Arthropoda; Hexapoda; Insecta; Pterygota; Neoptera; Endopterygota; Lepidoptera; Glossata; Ditrysia; Geometroidea; Geometridae; Larentiinae; *Eulithis*; *Eulithis prunata* (Linnaeus, 1758) (NCBI:txid934839).

## Background

The Phoenix, *Eulithis prunata*, is a moth in the family Geometridae distributed widely across northern Europe and Scandinavia, with scattered records from Eastern Europe and across Russia to Mongolia, China and Japan. There are also a few records and museum specimens from Canada and the United States, including some from the 1930s, suggesting occasional accidental importation ( GBIF Secretariat, 2022). The species is common across most of southern Britain, although never abundant, and is found most frequently in gardens or cultivated areas where the larval food plants grow ( Randle *et al.*, 2019; South, 1961).

*E. prunata* is larger than most members of the Geometridae found in Europe (wingspan 30–⁠35 mm) and has a chocolate-brown deeply-lobed cross band on the forewings, outlined in white. In northern Europe, the adult moth is on the wing primarily in July and August, and will come to light. Eggs are laid on the bark of the food plant, usually blackcurrant ( *Ribies nigrum*), redcurrant ( *R. rubrum*) or gooseberry ( *R. uva-crispa*), but embryonic development is delayed and the first instar larvae does not hatch until the following April. The larvae then feed on leaves of the currant or gooseberry bushes, pupating around June in a web spun between leaves ( Newman, 1869; South, 1961; Waring *et al.*, 2017).

A genome sequence for *E. prunata* will facilitate research into embryonic diapause and adaptations for host plant specificity, and will also contribute to the growing set of genomic resources for Lepidoptera.

### Genome sequence report

**Figure 1.**
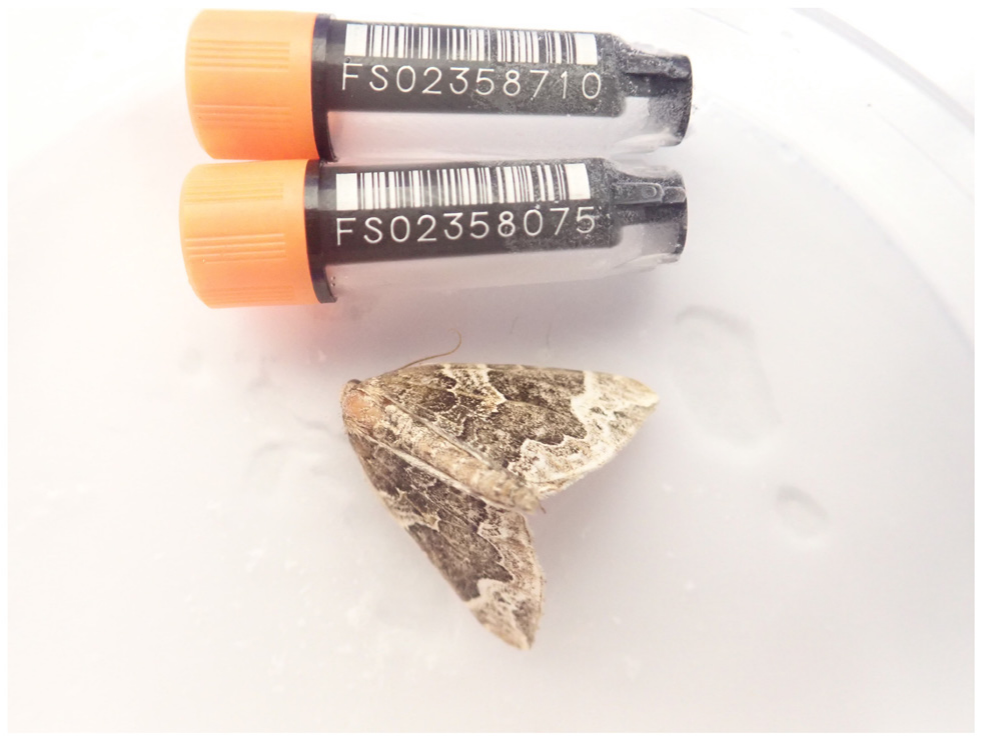
Photograph of the *Eulithis prunata* (ilEulPrun1) specimen used for genome sequencing.

The genome was sequenced from one male *Eulithis prunata* ( Figure 1) collected from Wytham Woods, Oxfordshire, UK (latitude 51.77, longitude –1.31). A total of 69-fold coverage in Pacific Biosciences single-molecule HiFi long reads and 177-fold coverage in 10X Genomics read clouds were generated. Primary assembly contigs were scaffolded with chromosome conformation Hi-C data. Manual assembly curation corrected one missing join, reducing the scaffold number by one.

**Figure 2.**
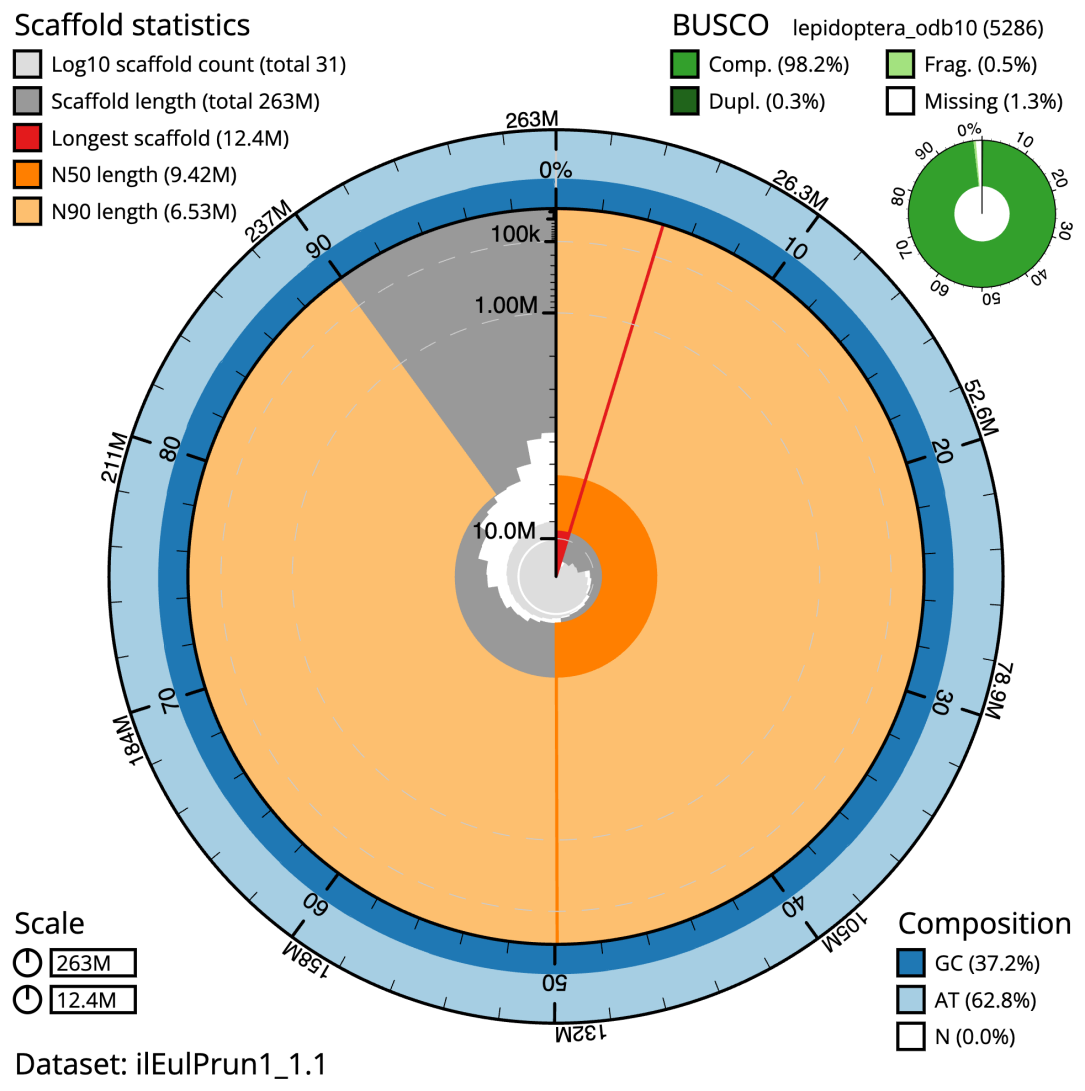
Genome assembly of *Eulithis prunata*, ilEulPrun1.1: metrics. The BlobToolKit Snailplot shows N50 metrics and BUSCO gene completeness. The main plot is divided into 1,000 size-ordered bins around the circumference with each bin representing 0.1% of the 263,147,802 bp assembly. The distribution of scaffold lengths is shown in dark grey with the plot radius scaled to the longest sequence present in the assembly (12,422,325 bp, shown in red). Orange and pale-orange arcs show the N50 and N90 scaffold lengths (9,415,980 and 6,527,104 bp), respectively. The pale grey spiral shows the cumulative scaffold count on a log scale with white scale lines showing successive orders of magnitude. The blue and pale-blue area around the outside of the plot shows the distribution of GC, AT and N percentages in the same bins as the inner plot. A summary of complete, fragmented, duplicated and missing BUSCO genes in the lepidoptera_odb10 set is shown in the top right. An interactive version of this figure is available at https://blobtoolkit.genomehubs.org/view/ilEulPrun1.1/dataset/ilEulPrun1_1.1/snail.

The final assembly has a total length of 263.1 Mb in 30 sequence scaffolds with a scaffold N50 of 9.4 Mb ( Table 1). The whole assembly sequence was assigned to 30 chromosomal-level scaffolds, representing 29 autosomes, and the Z sex chromosome. Chromosome-scale scaffolds confirmed by the Hi-C data are named in order of size ( Figure 2– Figure 5; Table 2). While not fully phased, the assembly deposited is of one haplotype. Contigs corresponding to the second haplotype have also been deposited. The mitochondrial genome was also assembled and can be found as a contig within the multifasta file of the genome submission.

**Table 1.** Genome data for *Eulithis prunata*, ilEulPrun1.1.

Project accession data		
Assembly identifier	ilEulPrun1.1	
Species	*Eulithis prunata*	
Specimen	ilEulPrun1	
NCBI taxonomy ID	934839	
BioProject	PRJEB46849	
BioSample ID	SAMEA7701309	
Isolate information	ilEulPrun1	
Assembly metrics *		*Benchmark*
Consensus quality (QV)	62	*≥ 50*
*k*-mer completeness	100%	*≥ 95%*
BUSCO **	C:98.2%[S:97.8%,D:0.3%], F:0.5%,M:1.3%,n:5,286	*C ≥ 95%*
Percentage of assembly mapped to chromosomes	100%	*≥ 95%*
Sex chromosomes	Z chromosome	*localised homologous pairs*
Organelles	Mitochondrial genome assembled	*complete single alleles*
Raw data accessions		
PacificBiosciences SEQUEL II	ERR6907890	
10X Genomics Illumina	ERR6688625–ERR6688628	
Hi-C Illumina	ERR6688629	
Genome assembly		
Assembly accession	GCA_918843925.1	
*Accession of alternate haplotype*	GCA_918843955.1	
Span (Mb)	263.1	
Number of contigs	35	
Contig N50 length (Mb)	9.0	
Number of scaffolds	30	
Scaffold N50 length (Mb)	9.4	
Longest scaffold (Mb)	12.4	
Genome annotation		
Number of protein-coding genes	16,023	
Number of gene transcripts	16,208	

* Assembly metric benchmarks are adapted from column VGP-2020 of “Table 1: Proposed standards and metrics for defining genome assembly quality” from ( Rhie *et al.*, 2021). ** BUSCO scores based on the lepidoptera_odb10 BUSCO set using v5.3.2. C = complete [S = single copy, D = duplicated], F = fragmented, M = missing, n = number of orthologues in comparison. A full set of BUSCO scores is available at https://blobtoolkit.genomehubs.org/view/ilEulPrun1.1/dataset/ilEulPrun1_1.1/busco.

**Figure 3.**
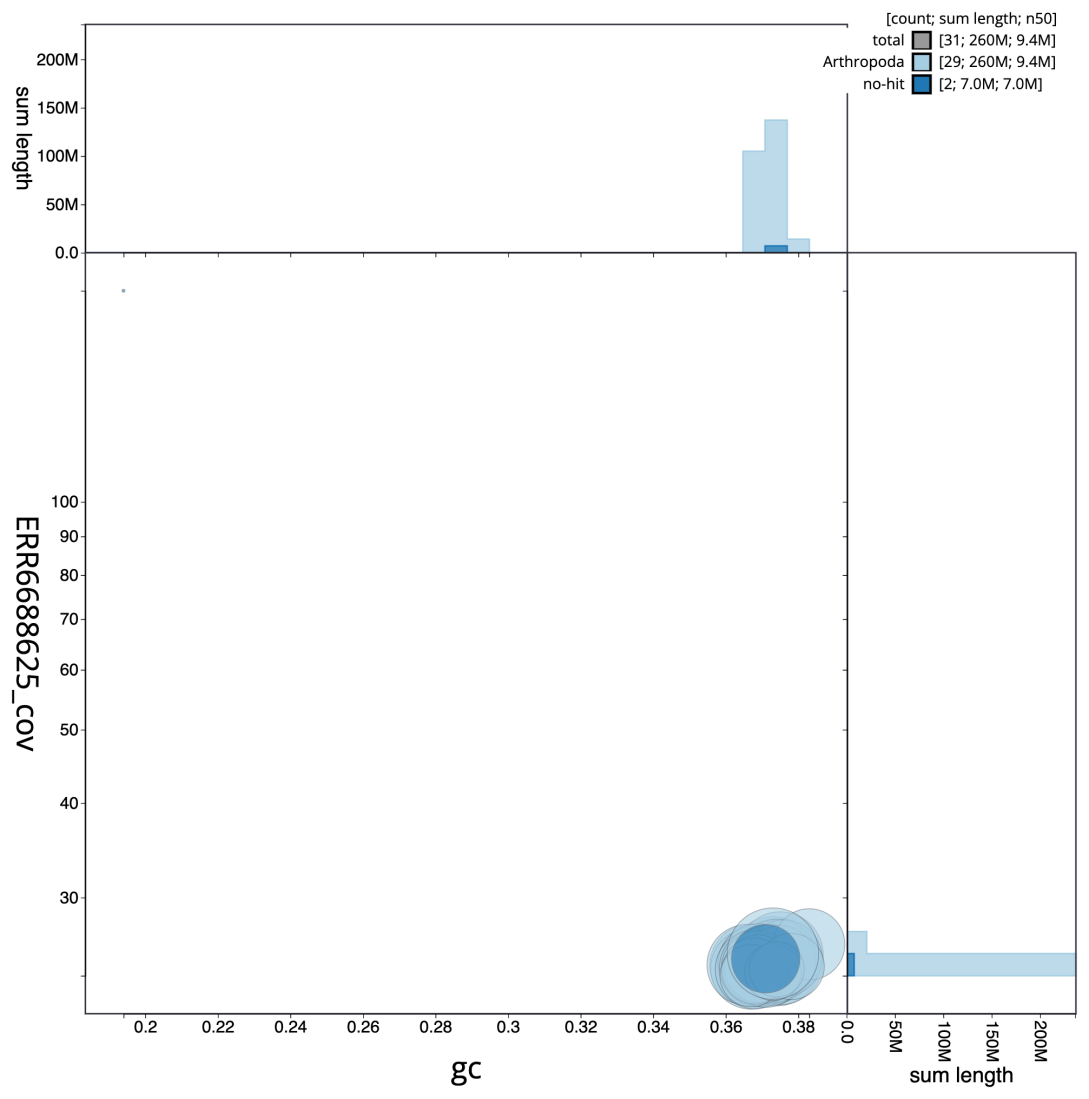
Genome assembly of *Eulithis prunata*, ilEulPrun1.1: GC coverage. BlobToolKit GC-coverage plot. Scaffolds are coloured by phylum. Circles are sized in proportion to scaffold length. Histograms show the distribution of scaffold length sum along each axis. An interactive version of this figure is available at https://blobtoolkit.genomehubs.org/view/ilEulPrun1.1/dataset/ilEulPrun1_1.1/blob.

**Figure 4.**
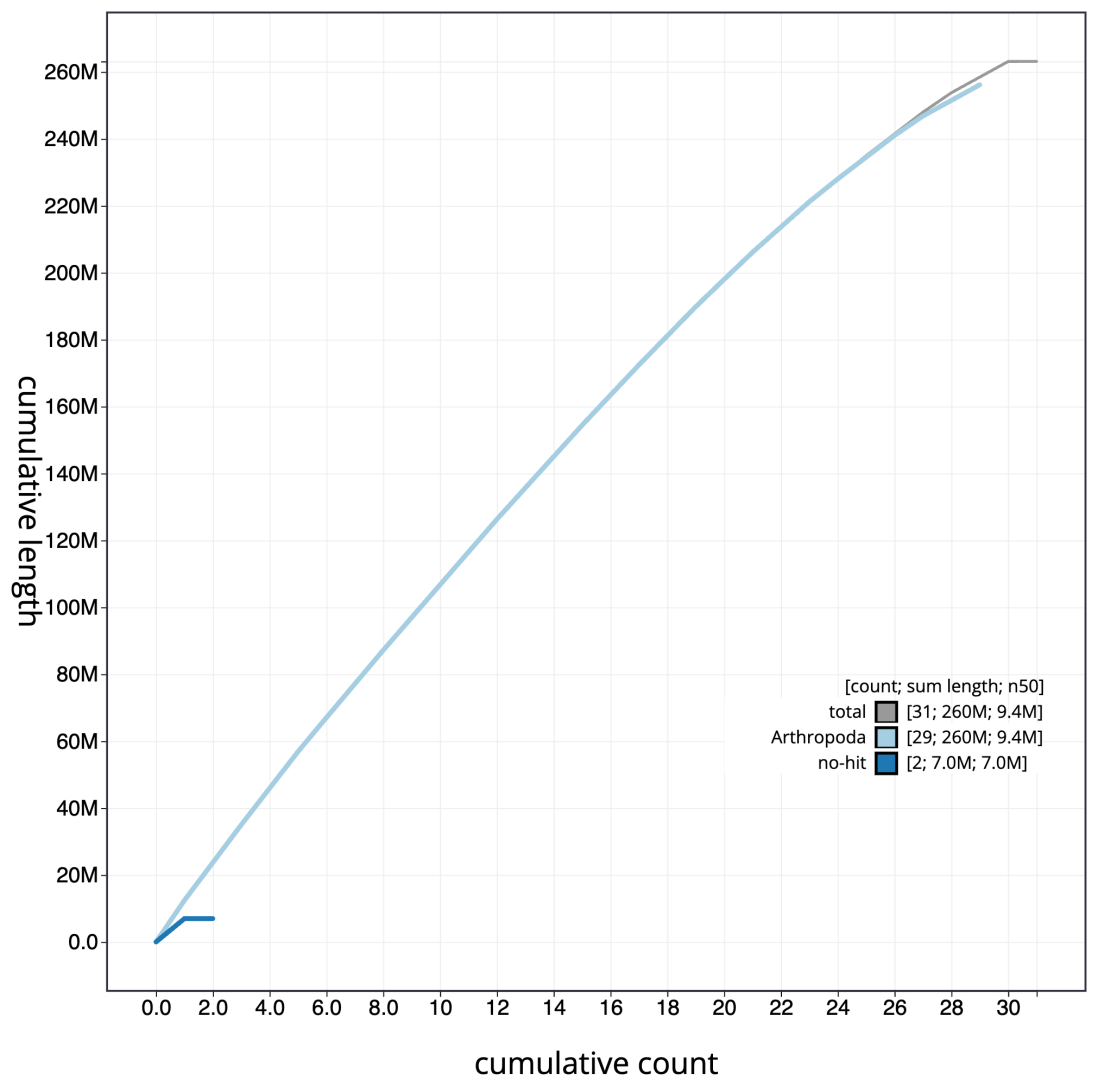
Genome assembly of *Eulithis prunata*, ilEulPrun1.1: cumulative sequence. BlobToolKit cumulative sequence plot. The grey line shows cumulative length for all scaffolds. Coloured lines show cumulative lengths of scaffolds assigned to each phylum using the buscogenes taxrule. An interactive version of this figure is available at https://blobtoolkit.genomehubs.org/view/ilEulPrun1.1/dataset/ilEulPrun1_1.1/cumulative.

**Figure 5.**
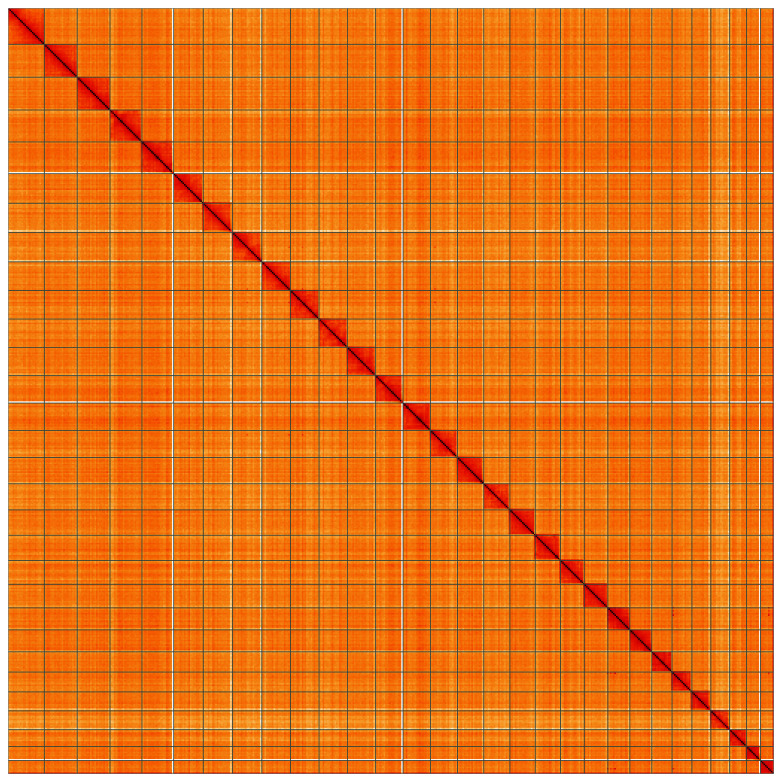
Genome assembly of *Eulithis prunata*, ilEulPrun1.1: Hi-C contact map. Hi-C contact map of the ilEulPrun1.1 assembly, visualised using HiGlass. Chromosomes are shown in order of size from left to right and top to bottom. An interactive version of this figure may be viewed at https://genome-note-higlass.tol.sanger.ac.uk/l/?d=JyygYVU5TBmpYhjfwKQK-Q.

**Table 2.** Chromosomal pseudomolecules in the genome assembly of *Eulithis prunata*, ilEulPrun1.

INSDC accession	Chromosome	Size (Mb)	GC%
OU974007.1	1	11.3	37.4
OU974008.1	2	11.28	37.3
OU974009.1	3	10.97	37.5
OU974010.1	4	10.91	37.5
OU974011.1	5	10.17	36.7
OU974012.1	6	10.08	36.9
OU974013.1	7	10.05	37
OU974014.1	8	9.87	37.4
OU974015.1	9	9.79	36.7
OU974016.1	10	9.77	36.6
OU974017.1	11	9.69	36.9
OU974018.1	12	9.42	37.2
OU974019.1	13	9.42	37.2
OU974020.1	14	9.29	36.8
OU974021.1	15	9.04	37.3
OU974022.1	16	8.97	36.9
OU974023.1	17	8.72	37.2
OU974024.1	18	8.69	37.6
OU974025.1	19	8.16	37.1
OU974026.1	20	8.11	36.9
OU974027.1	21	7.57	37.5
OU974028.1	22	7.54	38.3
OU974029.1	23	6.96	37.1
OU974030.1	24	6.81	36.9
OU974031.1	25	6.53	37.8
OU974032.1	26	6.46	36.8
OU974033.1	27	5.81	36.7
OU974034.1	28	4.78	37.3
OU974035.1	29	4.56	37.4
OU974006.1	Z	12.42	37.3
OU974036.1	MT	0.02	19.4

The estimated Quality Value (QV) of the final assembly is 62 with *k*-mer completeness of 100%, and the assembly has a BUSCO v5.3.2 completeness of 98.2% (single = 97.8%, duplicated = 0.3%), using the lepidoptera_odb10 reference set ( *n* = 5,286).

Metadata for specimens, spectral estimates, sequencing runs, contaminants and pre-curation assembly statistics can be found at https://links.tol.sanger.ac.uk/species/934839.

### Genome annotation report

The *Eulithis prunata* GCA_918843925.1 (ilEulPrun1.1) genome assembly was annotated using the Ensembl rapid annotation pipeline ( Table 1; https://rapid.ensembl.org/Eulithis_prunata_GCA_918843925.1/Info/Index). The resulting annotation includes 16,208 transcribed mRNAs from 16,023 protein-coding genes.

## Methods

### Sample acquisition and nucleic acid extraction

A male *Eulithis prunata* (ilEulPrun1) was collected from Wytham Woods, Oxfordshire (biological vice-county Berkshire), UK (latitude 51.77, longitude –1.31) on 25 June 2020. The specimen was taken from fen habitat by Douglas Boyes (University of Oxford) by netting. The specimen was identified by the collector and snap-frozen on dry ice.

DNA was extracted at the Tree of Life laboratory, Wellcome Sanger Institute (WSI). The ilEulPrun1 sample was weighed and dissected on dry ice with head and thorax tissue set aside for Hi-C sequencing. Abdomen tissue was disrupted using a Nippi Powermasher fitted with a BioMasher pestle *.* High molecular weight (HMW) DNA was extracted using the Qiagen MagAttract HMW DNA extraction kit. Low molecular weight DNA was removed from a 20 ng aliquot of extracted DNA using the 0.8X AMpure XP purification kit prior to 10X Chromium sequencing; a minimum of 50 ng DNA was submitted for 10X sequencing. HMW DNA was sheared into an average fragment size of 12–20 kb in a Megaruptor 3 system with speed setting 30. Sheared DNA was purified by solid-phase reversible immobilisation using AMPure PB beads with a 1.8X ratio of beads to sample to remove the shorter fragments and concentrate the DNA sample. The concentration of the sheared and purified DNA was assessed using a Nanodrop spectrophotometer and Qubit Fluorometer and Qubit dsDNA High Sensitivity Assay kit. Fragment size distribution was evaluated by running the sample on the FemtoPulse system.

### Sequencing

Pacific Biosciences HiFi circular consensus and 10X Genomics read cloud DNA sequencing libraries were constructed according to the manufacturers’ instructions. DNA sequencing was performed by the Scientific Operations core at the WSI on Pacific Biosciences SEQUEL II (HiFi) and Illumina NovaSeq 6000 (10X) instruments. Hi-C data were also generated from head and thorax tissue of ilEulPrun1 using the Arima2 kit and sequenced on the Illumina NovaSeq 6000 instrument.

### Genome assembly, curation and evaluation

The genome was assembled with Hifiasm ( Cheng *et al.*, 2021) and haplotypic duplication was identified and removed with purge_dups ( Guan *et al.*, 2020). One round of polishing was performed by aligning 10X Genomics read data to the assembly with Long Ranger ALIGN, calling variants with FreeBayes ( Garrison & Marth, 2012). The assembly was then scaffolded with Hi-C data ( Rao *et al.*, 2014) using SALSA2 ( Ghurye *et al.*, 2019). The assembly was checked for contamination and corrected using the gEVAL system ( Chow *et al.*, 2016) as described previously ( Howe *et al.*, 2021). Manual curation was performed using gEVAL, HiGlass ( Kerpedjiev *et al.*, 2018) and Pretext ( Harry, 2022). The mitochondrial genome was assembled using MitoHiFi ( Uliano-Silva *et al.*, 2022), which performed annotation using MitoFinder ( Allio *et al.*, 2020). To evaluate the assembly, MerquryFK was used to estimate *k*-mer completeness and consensus quality (QV) ( Rhie *et al.*, 2020). The genome was analysed, and BUSCO scores ( Manni *et al.*, 2021; Simão *et al.*, 2015) were generated within the BlobToolKit environment ( Challis *et al.*, 2020). Table 3 provides a list of software tool versions and sources.

**Table 3.** Software tools: versions and sources.

Software tool	Version	Source
BlobToolKit	4.0.7	https://github.com/blobtoolkit/blobtoolkit
BUSCO	5.3.2	https://gitlab.com/ezlab/busco
FreeBayes	1.3.1-17-gaa2ace8	https://github.com/freebayes/freebayes
gEVAL	N/A	https://geval.org.uk/
Hifiasm	0.15.3	https://github.com/chhylp123/hifiasm
HiGlass	1.11.6	https://github.com/higlass/higlass
Long Ranger ALIGN	2.2.2	https://support.10xgenomics.com/genome-exome/software/pipelines/latest/advanced/other-pipelines
Merqury	MerquryFK	https://github.com/thegenemyers/MERQURY.FK
MitoHiFi	2	https://github.com/marcelauliano/MitoHiFi
PretextView	0.2	https://github.com/wtsi-hpag/PretextView
purge_dups	1.2.3	https://github.com/dfguan/purge_dups
SALSA	2.2	https://github.com/salsa-rs/salsa

### Genome annotation

The BRAKER2 pipeline ( Brůna *et al.*, 2021) was used in the default protein mode to generate annotation for the *Eulithis prunata* assembly (GCA_918843925.1). in Ensembl Rapid Release.

### Ethics and compliance issues

The materials that have contributed to this genome note have been supplied by a Darwin Tree of Life Partner. The submission of materials by a Darwin Tree of Life Partner is subject to the Darwin Tree of Life Project Sampling Code of Practice. By agreeing with and signing up to the Sampling Code of Practice, the Darwin Tree of Life Partner agrees they will meet the legal and ethical requirements and standards set out within this document in respect of all samples acquired for, and supplied to, the Darwin Tree of Life Project. All efforts are undertaken to minimise the suffering of animals used for sequencing. Each transfer of samples is further undertaken according to a Research Collaboration Agreement or Material Transfer Agreement entered into by the Darwin Tree of Life Partner, Genome Research Limited (operating as the Wellcome Sanger Institute), and in some circumstances other Darwin Tree of Life collaborators.

## Data Availability

European Nucleotide Archive: *Eulithis prunata* (the phoenix). Accession number PRJEB46849; https://identifiers.org/ena.embl/PRJEB46849. ( Wellcome Sanger Institute, 2021) The genome sequence is released openly for reuse. The *Eulithis prunata* genome sequencing initiative is part of the Darwin Tree of Life (DToL) project. All raw sequence data and the assembly have been deposited in INSDC databases. Raw data and assembly accession identifiers are reported in Table 1. Members of the University of Oxford and Wytham Woods Genome Acquisition Lab are listed here: https://doi.org/10.5281/zenodo.4789928. Members of the Darwin Tree of Life Barcoding collective are listed here: https://doi.org/10.5281/zenodo.4893703. Members of the Wellcome Sanger Institute Tree of Life programme are listed here: https://doi.org/10.5281/zenodo.4783585. Members of Wellcome Sanger Institute Scientific Operations: DNA Pipelines collective are listed here: https://doi.org/10.5281/zenodo.4790455. Members of the Tree of Life Core Informatics collective are listed here: https://doi.org/10.5281/zenodo.5013541. Members of the Darwin Tree of Life Consortium are listed here: https://doi.org/10.5281/zenodo.4783558.
