# Supplementary material for: The genome sequence of the Phoenix, Eulithis prunata (Linnaeus, 1758)
Source: Wellcome Open Res. 2023 Apr 25;8:185. [Version 1] doi: 10.12688/wellcomeopenres.19371.1 (PMC10891429; doi:10.12688/wellcomeopenres.19371.1)
Supplement: Peer review history

# Reviewer report: Reviewer response for version 1

**DOI:** 10.21956/wellcomeopenres.21458.r64565

**Published:** 2025-01-13

**Authors:** Jeffrey Marcus

**Affiliations:** 1 University of Manitoba, Winnipeg, Manitoba, Canada

In this manuscript, the authors describe the sequencing and assembly of the *Eulithis prunata *genome using DNA from an adult male specimen collected in the UK. The primary genome sequence assembly includes proposed chromosomal pseudomolecule sequences for 30 autosomes, the Z sex chromosome, and a complete mitochondrial genome. Gene annotation of the assembly identified 16,023 putative protein-coding genes. On the whole, this is a useful contribution to the scientific literature, but please see my comments below:

 Some suggestions to the authors:

1. Method of Specimen identification: The individual who did the specimen identification was named, but keys/species descriptions consulted, or the morphological characters used for the identification have not been included in the manuscript, unless the morphological details included in the background section were used for this purpose (it’s not clear from the text).  I feel strongly that such details of how morphological identifications were conducted should be a requirement for publication.
2. For future work, I suggest that the researchers preferentially sequence the heterogametic sex when assembling genomes for previously unstudied species (in the case of Lepidoptera, the heterogametic sex is female), so that draft sequence assembles can be prepared for both sex chromosomes.

Are sufficient details of methods and materials provided to allow replication by others?

Partly

Is the rationale for creating the dataset(s) clearly described?

Yes

Are the datasets clearly presented in a useable and accessible format?

Yes

Are the protocols appropriate and is the work technically sound?

Yes

Reviewer Expertise:

Evolutionary biology of insects, phylogenomics

I confirm that I have read this submission and believe that I have an appropriate level of expertise to confirm that it is of an acceptable scientific standard, however I have significant reservations, as outlined above.

# Reviewer report: Reviewer response for version 1

**DOI:** 10.21956/wellcomeopenres.21458.r71995

**Published:** 2024-02-20

**Authors:** Muzafar Riyaz

**Affiliations:** 1 St Xavier's College, Palayamkottai, Tamil Nadu, India

The paper titled "The genome sequence of the Phoenix, Eulithis prunata" by Douglas Boyes and Peter W.H. Holland, along with their collaborators, provides the genome assembly of Eulithis prunata, commonly known as the Phoenix, a species of moth belonging to the Geometridae family. The authors have meticulously carried out genome sequencing, assembly, and annotation, offering a detailed insight into the genetic makeup of this fascinating moth.The inclusion of detailed methods and data availability information in the paper demonstrates transparency and facilitates reproducibility for future researchers interested in exploring this genome.

Are sufficient details of methods and materials provided to allow replication by others?

Yes

Is the rationale for creating the dataset(s) clearly described?

Yes

Are the datasets clearly presented in a useable and accessible format?

Yes

Are the protocols appropriate and is the work technically sound?

Yes

Reviewer Expertise:

Genomics; Phylogenomics; Moths

I confirm that I have read this submission and believe that I have an appropriate level of expertise to confirm that it is of an acceptable scientific standard.

# Reviewer report: Reviewer response for version 1

**DOI:** 10.21956/wellcomeopenres.21458.r72766

**Published:** 2024-02-16

**Authors:** María Cristina Castro

**Affiliations:** 1 University of Guanajuato, Guanajuato, Mexico

The article reports the *Eulithis prunata* **(the Phoenix; Arthropoda; Insecta; Lepidoptera; Geometridae). It is a merely descriptive article that reports the complete genome of this insect. There is no extra scientific contribution, only the description of this insect's genome components and mitochondria. The methodology used explains how the genome sequencing was carried out.

Are sufficient details of methods and materials provided to allow replication by others?

Yes

Is the rationale for creating the dataset(s) clearly described?

Yes

Are the datasets clearly presented in a useable and accessible format?

Yes

Are the protocols appropriate and is the work technically sound?

Yes

Reviewer Expertise:

Molecular biology of baculoviruses as insect biocontrol agents

I confirm that I have read this submission and believe that I have an appropriate level of expertise to confirm that it is of an acceptable scientific standard.
